# Correction: Ultrasensitive recognition of AP sites in DNA at the single-cell level: one molecular rotor sequentially self-regulated to form multiple different stable conformations

**DOI:** 10.1039/d2sc90066a

**Published:** 2022-04-29

**Authors:** Beidou Feng, Kui Wang, Yonggang Yang, Ge Wang, Hua Zhang, Yufang Liu, Kai Jiang

**Affiliations:** Henan Key Laboratory of Green Chemical Media and Reactions, Ministry of Education, Key Laboratory of Green Chemical Media and Reactions, Collaborative Innovation Center of Henan Province for Green Manufacturing of Fine Chemicals, Henan Key Laboratory of Organic Functional Molecules and Drug Innovation, School of Chemistry and Chemical Engineering, School of Environment, College of Physics and Materials Science, Henan Normal University Xinxiang 453007 China zhanghua1106@163.com; Xinxiang Medical University Xinxiang 453000 P. R. China

## Abstract

Correction for ‘Ultrasensitive recognition of AP sites in DNA at the single-cell level: one molecular rotor sequentially self-regulated to form multiple different stable conformations’ by Beidou Feng *et al.*, *Chem. Sci.*, 2019, **10**, 10373–10380, DOI: 10.1039/C9SC04140K.

The authors regret that the descriptions of the DNA sequences in the original legend of Fig. 2 were inappropriate. The correct descriptions in the legend of Fig. 2 are presented below, and the detailed DNA sequences were added in page S2 of the revised ESI file associated with the original article (DOI: 10.1039/C9SC04140K) and are copied below.

**Fig. 2:**
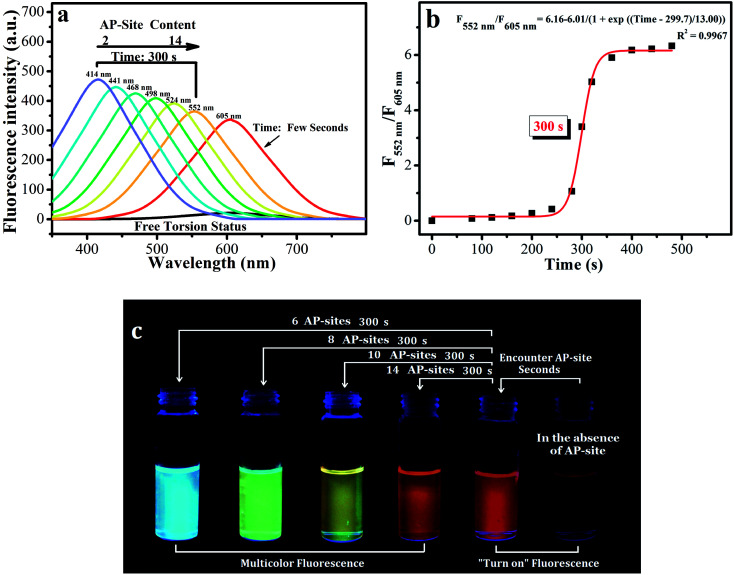
(a) Spectral data of d1-BMN (3.0 mM) for AP sites (2, 4, 6, 8, 10 and 14 AP sites/first 20 bp from 5′ to 3′ of DNA (60 bp), according to the given DNA sequence) in PBS buffer (pH = 7.4) at different reaction times (0, few seconds and 300 s). All DNA sequences with AP sites were synthesized by Thermo Fisher Scientific; the DNA sequence information is listed in the Procedures section of the ESI.† Excitation wavelength = 400 nm and 354 nm. (b) The reaction time of d1-BMN (3.0 mM) for AP sites (14 AP sites/first 20 bp from 5′ to 3′ of DNA (60 bp)) in PBS buffer (pH = 7.4). (c) d1-BMN emitting multicolor fluorescence, visible to the naked eye, for different contents of AP sites (6, 8, 10 and 14 AP sites/first 20 bp from 5′ to 3′ of DNA (60 bp)) in PBS buffer (pH = 7.4) at different reaction times (0, few seconds and 300 s).^23^ The number of AP sites in the DNA sequence was quantitatively detected using an AP-site counting kit (Dojindo, Japan, see ESI†).

**Detailed DNA sequences (also included in the revised ESI):**

DNA sequence: TTCTAGGCTCCTAGGACCCC TTCTAGGCTCCTAGGACCCC TTCTAGGCTCCTAGGACCCC;

2 AP sites in DNA sequence: TTCTAGG(RDG)(RDG)CCTAGGACCCC TTCTAGGCTCCTAGGACCCC TTCTAGGCTCCTAGGACCCC;

4 AP sites in DNA sequence: TTCTAG(RDG)(RDG)(RDG)(RDG)CTAGGACCCC TTCTAGGCTCCTAGGACCCC TTCTAGGCTCCTAGGACCCC;

6 AP sites in DNA sequence: TTCTA(RDG)(RDG)(RDG)(RDG)(RDG)(RDG)TAGGACCCC TTCTAGGCTCCTAGGACCCC TTCTAGGCTCCTAGGACCCC;

8 AP sites in DNA sequence: TTCT(RDG)(RDG)(RDG)(RDG)(RDG)(RDG)(RDG)(RDG)AGGACCCC TTCTAGGCTCCTAGGACCCC TTCTAGGCTCCTAGGACCCC;

10 AP sites in DNA sequence: TTC(RDG)(RDG)(RDG)(RDG)(RDG)(RDG)(RDG)(RDG)(RDG) (RDG)GGACCCC TTCTAGGCTCCTAGGACCCC TTCTAGGCTCCTAGGACCCC;

14 AP sites in DNA sequence: TTC(RDG)(RDG)(RDG)(RDG)(RDG)(RDG)(RDG)(RDG)(RDG)(RDG) (RDG)(RDG)(RDG)(RDG)CCC TTCTAGGCTCCTAGGACCCC TTCTAGGCTCCTAGGACCCC

In addition, the authors regret that the abbreviation and the naming of compounds were incorrect in the original article and ESI. The correct name for the compound abbreviated HDM in Scheme S1 should be (*R*)-3,5-dimethoxy-4-oxopentanal. The correct version of Scheme S1 is presented below.

**Scheme 1:**
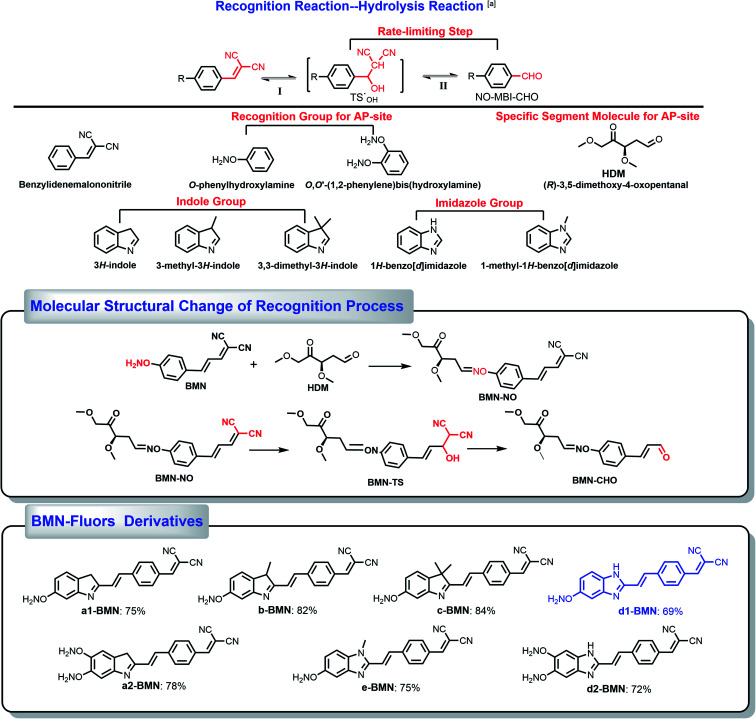
Derivative molecules. The hydrolysis transformation of 2-(4-vinylbenzylidene)malononitrile derivatives.

(*E*)-2-(3-(4-(aminooxy)phenyl)allylidene)malononitrile (Fig. 3a,b and Scheme S1) was used as the simplest molecular fragment to better explain the structural changes during the recognition process. Its correct abbreviation is BMN, which has been corrected in Fig. 3a, b and Scheme S1. The caption of Fig. 3a has also been updated. In addition, the corresponding ^1^H NMR titration spectra have been included in the revised ESI file associated with the original article (DOI: 10.1039/C9SC04140K).

**Fig. 3:**
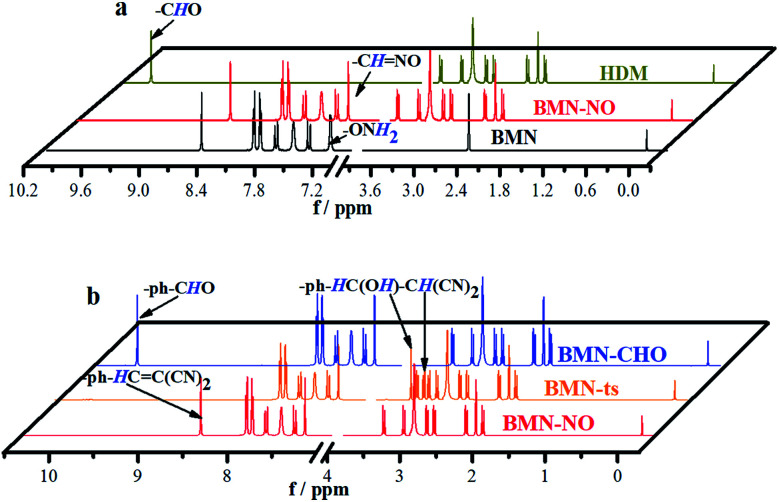
(a and b) ^1^H NMR spectroscopy in DMSO-d_6_ of different compounds during the recognition process. Black line: BMN; dark yellow line: HDM; red line: BMN-NO. (b) NMR spectra monitoring the molecular structure changes during the recognition process in Fig. 1a and c. Red line: BMN-NO, orange line: BMN-ts, blue line: BMN-CHO.

The Royal Society of Chemistry apologises for these errors and any consequent inconvenience to authors and readers.

## Supplementary Material

